# Histamine H4 receptor antagonism diminishes existing airway inflammation and dysfunction via modulation of Th2 cytokines

**DOI:** 10.1186/1465-9921-11-86

**Published:** 2010-06-24

**Authors:** Jeffery M Cowden, Jason P Riley, Jing Ying Ma, Robin L Thurmond, Paul J Dunford

**Affiliations:** 1 Immunology, Johnson & Johnson Pharmaceutical Research & Development, L.L.C. San Diego, California, USA

## Abstract

**Background:**

Airway remodeling and dysfunction are characteristic features of asthma thought to be caused by aberrant production of Th2 cytokines. Histamine H_4 _receptor (H_4_R) perturbation has previously been shown to modify acute inflammation and Th2 cytokine production in a murine model of asthma. We examined the ability of H_4_R antagonists to therapeutically modify the effects of Th2 cytokine production such as goblet cell hyperplasia (GCH), and collagen deposition in a sub-chronic model of asthma. In addition, effects on Th2 mediated lung dysfunction were also determined.

**Methods:**

Mice were sensitized to ovalbumin (OVA) followed by repeated airway challenge with OVA. After inflammation was established mice were dosed with the H_4_R antagonist, JNJ 7777120, or anti-IL-13 antibody for comparison. Airway hyperreactivity (AHR) was measured, lungs lavaged and tissues collected for analysis.

**Results:**

Therapeutic H_4_R antagonism inhibited T cell infiltration in to the lung and decreased Th2 cytokines IL-13 and IL-5. IL-13 dependent remodeling parameters such as GCH and lung collagen were reduced. Intervention with H_4_R antagonist also improved measures of central and peripheral airway dysfunction.

**Conclusions:**

These data demonstrate that therapeutic H_4_R antagonism can significantly ameliorate allergen induced, Th2 cytokine driven pathologies such as lung remodeling and airway dysfunction. The ability of H_4_R antagonists to affect these key manifestations of asthma suggests their potential as novel human therapeutics.

## Background

The pathology of chronic asthma is characterized by inflammation and remodeling of airway tissues. As a result of repeated inflammatory insults to the lung, smooth muscle thickening, mucin secretion and airway hyperreactivity may develop [1]. The current consensus as to the etiology of allergic asthma defines it is an aberrant T-helper-2 (Th2) type response to environmental allergens characterized by overproduction of IL-4, IL-5, and IL-13 which are critical in maintaining an ongoing IgE-mediated, eosinophilic inflammation [2].

Polarization of naïve Th0 cells to the Th2 and other T helper sub-sets may be differentially controlled at the level of the interaction between dendritic cells (DCs) and antigen-specific T cells. Such interaction can be directed by a variety of cytokines, chemokines, toll-ligands and biogenic amines, such as histamine. These are released at sites where antigen is encountered or presented and may sequentially modulate the dendritic cell and subsequent T helper phenotypes [3].

Histamine has long been thought of as an important mediator of asthma due to its ability to recapitulate symptoms of asthma, such as bronchoconstriction, and measured levels being correlated with asthma severity [4,5]. However, the inefficacy of traditional antihistamines, H_1 _receptor (H_1_R) antagonists, has lead to the belief that it is not a viable target for asthma therapy.

Recently, a fourth receptor for histamine, the histamine H_4 _receptor (H_4_R) has been identified as a potential modulator of dendritic cell activation and T cell polarization and to have a distinct pharmacological profile from H_1_R [6]. H_4_R is functionally expressed on many cell types intimately associated with the pathology of asthma, such as eosinophils, basophils, mast cells, dendritic cells and CD8+ T cells, as recently reviewed [7]. Selective antagonism or gene knockout of H_4_R has been demonstrated to diminish allergic lung inflammation in a mouse model, with specific reduction of Th2-type cytokines identified in bronchoalveolar lavage fluid (BALF) and from draining lymph node cultures. Notably, a profound reduction in Th2 polarization and the production of the effector Th2 cytokine, IL-13, was observed [6].

IL-13 is thought to be a critical mediator of allergic asthma, with genetic and pharmacological evidence supporting its involvement in the development of airway hyperreactivity (AHR) and the development of chronic asthma and remodeling phenotypes [8,9]. As such, numerous approaches to blocking increased IL-13 in asthma are being evaluated, with emphasis on IL-13 neutralizing antibodies and soluble receptors, but the identification of oral, small molecule inhibitors of IL-13 would have obvious advantages. We therefore sought to examine whether the previously reported modulation of IL-13, and other Th2 cytokines, by H_4_R antagonists could have a meaningful therapeutic effect on inflammation, remodeling and airway dysfunction in a sub-chronic model of allergic lung inflammation in the mouse

## Methods

### Mice

BALB/c female mice (6-8 weeks old) were from Charles River Laboratories. All mice were maintained under specific pathogen-free conditions and maintained on an OVA-free diet with free access to food and water. All experimental animals used in this study were under a protocol approved by the Institutional Animal Care and Use Committee of Johnson & Johnson Pharmaceutical Research & Development, L.L.C.

Rat Anti-Mouse IL-13, CNTO 134, (IgG2a isotype) was kindly provided by Dr Wil Glass (Centcor Inc, Malvern, PA). JNJ 7777120 was synthesized in the laboratories of Johnson & Johnson Pharmaceutical Research & Development, L.L.C., as previously described [10]. It is a selective H_4_R antagonist with a K_i _at the mouse H_4_R of 5 Nm [11]. Compound was prepared in solution of 20% hydroxypropyl- beta- cyclodextran (HPCD), w/v in H_2_O, at various concentrations.

### Induction of sub-chronic airway inflammation

Mice were immunized intra-peritoneally (i.p.) with 10 μg OVA (Sigma-Aldrich, St. Louis, MO) in PBS and Inject Alum (Pierce, Rockford, IL) mixed 1:1 on day 1 and boosted in the same way on day 8. On day 22, 29, 36, 43, 50, and 57, mice received an intranasal (i.n.) challenge with 50 μl of PBS or 100 μg of OVA in PBS (2 mg/ml) under isoflourane anesthesia. Anti-mouse IL-13 mAb (weekly i.v. 500 μg) or H_4_R antagonist JNJ 7777120 (once daily, per os.) treatment was initiated on day 36 once inflammation had already developed and continued through day 58. Agents were administered 1 h prior to each i.n. challenge. Mice were sacrificed on day 30 (to confirm existing inflammation) or day 59 with a terminal dose of 100 mg/kg sodium pentobarbital. Serum was obtained from mice and lungs sampled for inflammation parameters as described below.

### Bronchoalveolar lavage (BAL)

Following euthanasia BAL samples were obtained, processed and inflammatory cells counted as previously described [6]. Supernatants were immediately frozen for subsequent cytokine level analysis by ELISA, as described below.

### T cell proliferation in draining lymph nodes

Peribronchiolar lymph nodes (PBLN) were collected and pooled. A single cell suspension was prepared and cultured in 96 wells (0.4 million cells/per well) with or without 100 μg of OVA. After 96 h, 1°C of [^3^H] Thymidine was added for 18 hours. Cells were collected on a filter and [^3^H] incorporation quantified. Supernatants from non-thymidine treated, parallel 96 h cultures were frozen for subsequent cytokine level analysis by ELISA, as described below.

### Enzyme-Llinked immunosorbent assays (ELISAs)

Cytokines, IL-4, IL-5 and IL-13 levels were determined in BALF, homogenized lung preparations and in PBLN culture supernatants by ELISA (R&D Systems, Minneapolis, MN). Chemokines CCL3, CCL5 and CCL11 were similarly measured in lung homogenates. All assays followed manufacturers' directions.

### Protein concentration

Tissue was homogenized in PBS using a Fast-Prep homogenizer (Thermo Savant, Holbrook, NY) and protein content assayed by BCA assay (Pierce, Rockford, IL) as per the manufacturers' instructions.

### Total collagen

Free collagen was measured from the supernatants of homogenized lung tissue in 1 ml PBS using the Sircol, dye-binding collagen assay kit (Biocolor, Belfast, UK) according to the manufacturer's instructions.

### Histology

Following BAL, lungs were fixed with 10% formalin under constant pressure of 15-cm water. After fixation, lungs were dehydrated and embedded in paraffin by routine methods parahilar sagittal sections were obtained. Serial sections were stained with hematoxylin and eosin (H&E) or periodic acid Schiff (PAS)/alcian blue (counterstained with hematoxylin).

For CD3+ (IHC) staining, slides were deparaffinized and hydrated in PBS followed by blocking the endogenous peroxide with 3% hydrogen peroxide. To avoid nonspecific reaction with secondary antibody, slides were pretreated with 10% normal donkey serum before incubation with CD3. The CD3+ primary antibody used in this study was goat anti-CD3 (2 μg/ml) at a dilution of 1:100 (Santa Cruz Biotechnology, Inc. cat. No. sc-1127 and the secondary antibody used was donkey anti-goat biotinlated IgG (0.5 μg/ml) (Chemicon International, Inc. cat. No. AP180B) at a dilution of 1:2000. Normal goat IgG was used as negative controls. The immunoreactivities were visualized by ABC reagents (Vector, Burlingame, cat. No. PK-6100) and diaminobezidine (Research Genetic, Cat. No. 750118) followed by counterstaining with hematoxylin. CD3+ IHC was quantified by counting five independent hot fields around the main segmental bronchus.

For semiquantitative analysis of GCH, sections were analyzed morphometrically using Simple PCI image analysis software (Compix Inc, PA). PAS-stained sections were thresholded by color identification to measure only the area of mucin content. Mucin content was normalized to the diameter of each airway. At least three separate airways from each specimen were measured.

### Measurement of airway hyperreactivity

Airway hyperreactivity was induced in mice using a previously described protocol [6]. Animals received anti-mouse IL-13 mAb once one day prior to ovalbumin challenge (i.v. 500 μg) or H_4_R antagonist JNJ 7777120, 20 mg/kg (b.i.d. p.o.) prior to and 8 hours after each of four daily challenges. Twenty four hours after the fourth ovalbumin challenge lung function measurements were assessed using a computer controlled small animal ventilator (Scireq, Montreal, Canada).

Mice were anesthetized using intra-peritoneal injection of 100 mg/kg sodium pentobarbital (Euthasol, ANADA#2). Mechanical respiration on the flexivent was immediately initiated using a tidal volume of 9 ml/kg at a rate of 150 breaths/min, with a positive end-expiratory pressure of 3 cm H_2_O. Animals were allowed to acclimate to the respirator for approximately two minutes to establish a stable baseline. At this time airway responses were measured subsequent to aerosolized doses of methacholine, 0 mg/ml, 25 mg/ml and 50 mg/ml, using forced oscillation techniques. The resultant pressure and flow data were fit into a constant phase model as previously described [12] and analyzed to compare drug-treated groups with vehicle-treated animals. The mean of 12 sets of data after each aerosol challenge was analyzed for individual animals.

Similar to other studies assessing forced oscillatory mechanics we confined our analysis to: R_N _(Newtonian resistance), which assesses the flow resistance of the conducting airways; G (tissue damping), which reflects tissue resistance and H (tissue elastance), which reflects the tissue rigidity [13].

### Statistical analysis

One-way analysis of variance, followed by Dunnett's multiple comparison test, were performed where indicated. In all cases the *P *value was calculated based on the difference between the vehicle treated controls and respective treatment group in each study. A two-way analysis of variance, with Bonferroni post-test was performed for airway hyperreactivity measurements. The error bars shown represent the SEM. In all cases the experiments were repeated two to three times with similar results and representative data are shown.

## Results

### H_4_R antagonism therapeutically inhibits lung and BAL Th2 cytokines

**Figure 1:**
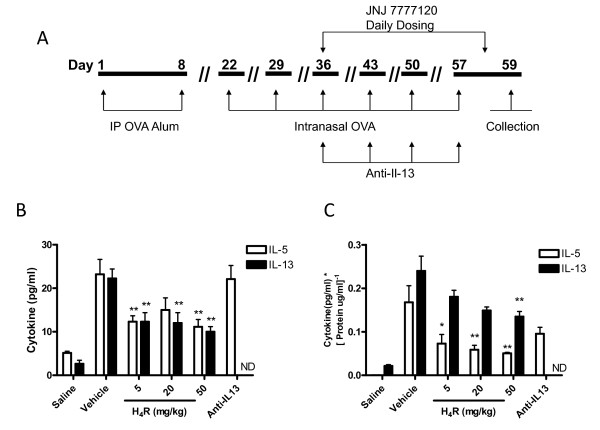
**An H_4_R antagonist therapeutically decreases Th2 associated cytokines from BAL fluid and lung tissue**. An H_4_R antagonist therapeutically decreases Th2 associated cytokines from BAL fluid and lung tissue in a sub-chronic model of allergic airway inflammation. (A) Model schematic (B) Cell free BAL fluid from vehicle, anti-IL-13 and JNJ 7777120 treated mice (5, 20 and 50 mg/kg) was assayed for the indicated cytokines by using ELISA. (C) Lung homogenates from the same animals were assayed for cytokine content and corrected for total protein. n = 8-10. Significance of each treatment group compared to control vehicle-treated animals is as follows: * *P *< 0.05; ** *P *< 0.01; ND = Not determined.

To examine the utility of H_4_R antagonists dosed in a therapeutic regimen we utilized a sub-chronic model of allergic airway inflammation, [14] and (Fig 1A), in which dosing of JNJ 7777120 or anti-IL-13 antibody were only initiated after elicitation of inflammation through two intranasal ovalbumin challenges in previously sensitized animals. Confirmation of inflammation by measurement of airway inflammation and Th2 cytokine induction was confirmed prior to the commencement of treatment (Table 1).

**Table 1:** Lung Inflammatory parameters at Commencement of Drug Treatment

		BALF Cells (×10^6^)				Lung Cytokines (pg/μg protein)	
		
	WBCs	Macs	Eos	Neuts	Lymph	IL-5	IL-13
Saline	0.35 ± 0.05	0.35 ± 0.05	0	0	0	BLLOQ	0.01 ± 0.003
OVA	1.85 ± 0.10	0.79 ± 0.06	0.48 ± 0.07	0.57 ± 0.05	0.005 ± 0.004	0.35 ± 0.05	1.24 ± 0.15

Definition of Abbreviations: BALF = bronchoalveolar lavage fluid; BLLOQ = below lower limit of quantification; eos = eosinophils; OVA = ovalbumin; WBCs = white blood cells. Values represent mean ± SEM

After therapeutic treatment with the H_4_R antagonist, significantly reduced levels of IL-13 were detected compared to vehicle treatment in the BALF (vehicle, 22.3 ± 2.2 pg/ml versus 5 mg/kg H_4_R, 12.3 ± 2.1 pg/ml *P *< 0.01) (Fig 1B), and in the tissue (vehicle, 0.24 ± 0.03 pg/ml versus 5 mg/kg H_4_R, 0.12 ± 0.01 pg/μg, *P *< 0.01) (Fig 1C). Unfortunately, the nature of the anti-IL-13 antibody made it impossible to distinguish IL-13 that has been neutralized from active form using the ELISA assay, so a comparison of IL-13 levels between vehicle and anti-IL-13 treated groups was not possible. Levels of IL-5 were also significantly reduced in BALF (vehicle, 23.2 ± 3.4 pg/μg tissue versus 5 mg/kg H_4_R, 12.3 ± 1.3 pg/μg tissue *P *< 0.01) and in lung homogenate (vehicle, 0.17 ± 0.04 pg/μg versus 5 mg/kg H_4_R, 0.05 ± 0.003 pg/μg, *P *< 0.01) after H_4_R antagonist treatment. Anti-IL-13 had no effect on IL-5 levels in either media.

### Inhibition of draining lymph node T cell proliferation and cytokine production

**Figure 2:**
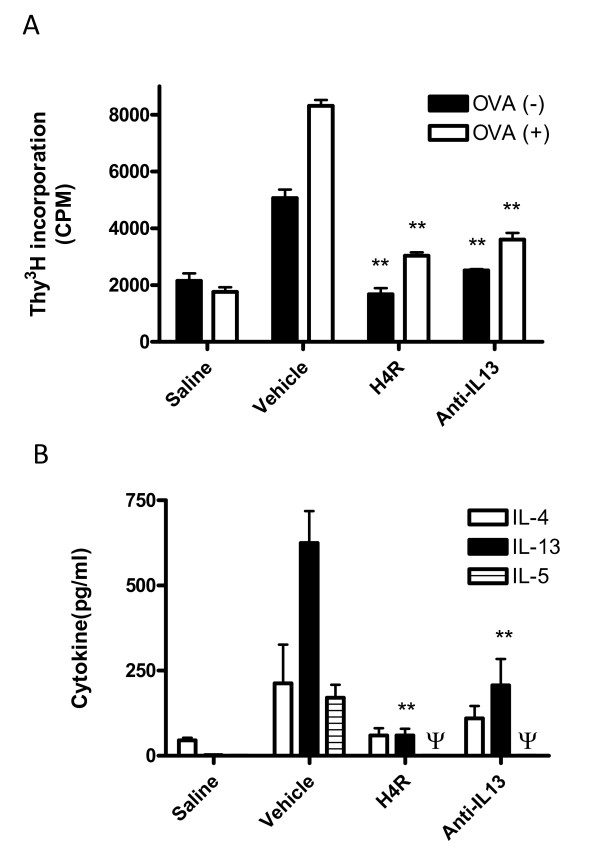
**H_4_R antagonism decreases antigen-specific lymph node proliferation and cytokine production**. (A) PBLN from vehicle, JNJ 7777120 (20 mg/kg) and anti-IL-13 treated animals were cultured with and without the addition of ovalbumin. Proliferation was determined by the measurement of incorporated ^3^H thymidine. (B) Supernatants from parallel lymph node cultures treated with ovalbumin were assayed for IL-4, IL-5 and IL-13 by ELISA. Lymph nodes were pooled from 8-10 animals per group and assayed in quadruplicate. Significance of each treatment group compared to control vehicle-treated animals is as follows: * *P *< 0.05, ** *P *< 0.01, *** *P *< 0.001, Ψ < 15 pg/ml, the lower limit of quantification in this assay.

The effect of H_4_R antagonist treatment on underlying T cell responses in the model was determined by examining draining lymph node proliferation and cytokine production in response to antigen specific stimulation. T cells from both H_4_R antagonist (20 mg/kg) and anti-IL-13 treated groups (3035 ± 209 CPM and 3601 ± 117 CPM, *P *<0.01, respectively versus vehicle, 8316 ± 235 CPM) had decreased proliferation upon re-stimulation with antigen (Fig 2A). In addition, levels of IL-5 and IL-13 in OVA-stimulated culture supernatants were significantly decreased by H_4_R antagonist and anti-IL-13 treatment. (Fig 2B). The levels of IL-5 from lymph nodes of treated animals were below the level of quantification, which was 15 pg/ml. There was also trend towards a reduction in IL-4 levels. This last finding may explain the observed significant reduction in serum ova-specific IgE after JNJ 7777120 treatment (see figure S1, additional file 1).

### H_4_R antagonism reduces lung tissue and lumenal inflammation

**Figure 3:**
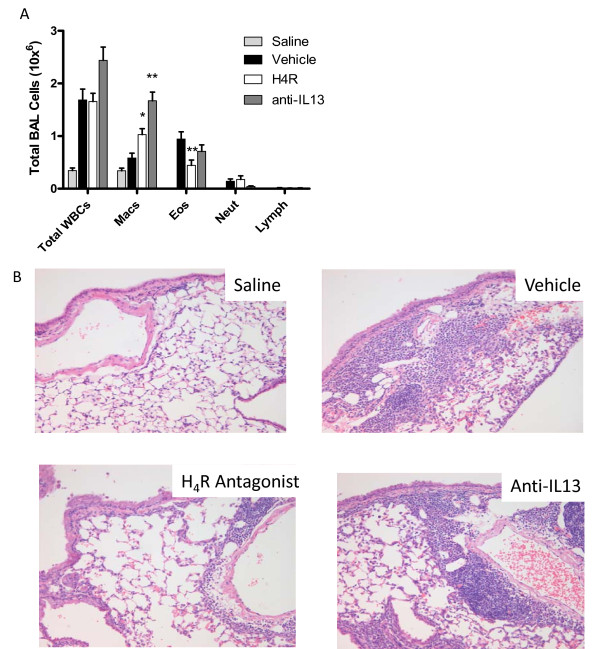
**An H_4_R antagonist inhibits sub-chronic allergic airway inflammation in Balb/C mice**. (A) The total number of white blood cells (WBCs) and differential cell count for eosinophils, monocytes neutrophils and lymphocytes were calculated from BAL fluid collected after the final OVA challenge. JNJ 7777120 was dosed at 20 mg/kg. n = 8-10 (B) Lung histology, hematoxylin and eosin stain (x200 magnification). Significance of each treatment group compared to control vehicle-treated animals is as follows: * *P *< 0.05, ** *P *< 0.01.

**Figure 4:**
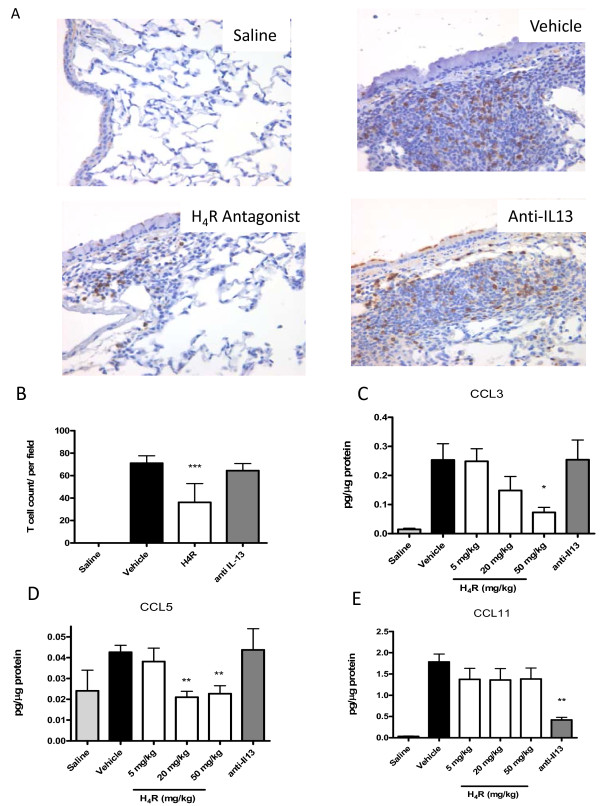
**An H_4_R antagonist inhibits T cell chemokines and T cell influx in to allergen challenged lungs**. Lungs from vehicle, JNJ 7777120 (20 mg/kg) and anti-IL-13 treated animals were sectioned and stained with anti-CD3+ antibody to highlight T cells. (A) Lung histology with CD3+ stain (400× magnification). n = 4. (B) CD3 + cells were quantified by a blinded observer. (C-E) Lung homogenates from the same animals were assayed for chemokine content and corrected for total protein. n = 8-10. Significance of each treatment group compared to control vehicle-treated animals is as follows: * *P *< 0.05, ** *P *< 0.01, *** *P *< 0.001

Leukocyte influx into the lung lumen was assessed by lavage 24 hours after the sixth weekly challenge of OVA. Eosinophilic inflammation routinely peaks at 48 hours after an allergen challenge in mice, yet we sampled at 24 hours to allow for the concomitant assessment of cytokines. Accordingly, a somewhat mixed eosinophil and neutrophil population was observed at this time point (Table 1 and Fig 3A). Treatment with the H_4_R antagonist at 20 mg/kg, initiated on top of an existing inflammation, significantly reduced the number of eosinophils in the lavage fluid by 61% (vehicle, 0.94 ± 0.14 × 10^6 ^cell/ml versus H_4_R, 0.37 ± 0.7 × 10^6 ^cell/ml *P *< 0.01), however treatment with anti-IL-13 antibody failed to statistically reduce eosinophil influx (Fig. 3A). Similar trends in the reduction of inflammation were observed in histological sections of the lung (Fig 3B), and as measured by a blinded pathological score (data not shown). Specific quantification of CD3 + cell influx from immunohistochemical histology (Fig 4A) revealed a significant, 49% decrease in OVA challenged animals when dosed with H_4_R antagonist (vehicle, 71 ± 3 T cells/field versus H_4_R, 36.2 ± 7.5 T cells/field, *P *< 0.001) but not when dosed with anti-IL-13 (Fig 4B). Intranasal administration of PBS to OVA sensitized animals failed to cause leukocyte recruitment to the lungs indicating the response to ovalbumin was antigen specific.

### H_4_R antagonism inhibits T cell attractant chemokines in lung

The mechanism by which H_4_R antagonism might reduce T cell infiltration in to the lung was examined by the measurement of chemokines in lung homogenates. From a range of chemokines measured, corrected for total protein levels, only CCL3, CCL5 and CCL11 were modulated significantly by H_4_R antagonism or ani-IL-13 treatment. The potent T cell chemoaatractants, CCL3 (Fig 4C) and CCL5 (Fig 4D) were significantly and dose dependently attenuated by H_4_R antagonist treatment, while CCL11 (eotaxin) was unchanged (Fig 4E). Conversely, anti-IL-13 treatment significantly inhibited CCL11 production, with no effect on CCL3 or CCL5.

**Figure 5:**
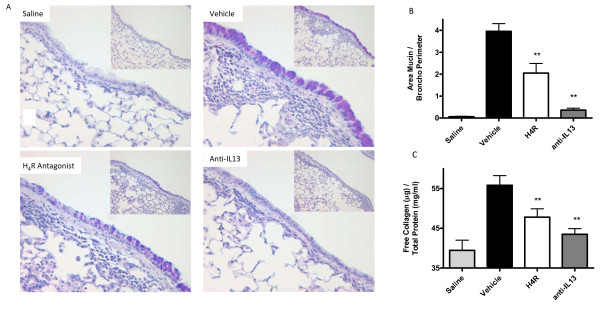
**An H_4_R antagonist reduces mucus content and free collagen in the airways of allergen challenged lungs**. Lungs from vehicle, JNJ 7777120 (20 mg/kg) and anti-IL-13 treated animals were sectioned and stained with alcian blue/PAS. (A) Lung histology with Alcian blue/PAS stain. 400× magnification (200× inset) n = 4. (B) Area of mucin staining per length of airway epithelium was calculated using image analysis software. (C) H_4_R antagonism inhibits collagen deposition in allergen challenged lungs. Lungs from vehicle, JNJ 7777120 (20 mg/kg) and anti-IL-13 treated animals were lavaged and resulting BALF was analyzed for free collagen levels. n = 3-8. Significance of each treatment group compared to control vehicle-treated animals is as follows: ** *P *< 0.01.

A comparable study, in which H4R antagonist, but not anti-IL-13 was examined revealed an additional dose dependent and significant inhibition of CCL17 (TARC) production via H4R antagonism (see figure S2, additional file 2).

### H_4_R antagonism suppresses goblet cell hyperplasia

In addition to investigating the anti-inflammatory effects of H_4_R antagonism, it was important to assess whether its modulation of Th2 cytokines could have meaningful effects on allergen induced airway structural changes. Consequently, an Alcian Blue/PAS stain was used to identify mucin in the airway epithelium of lung tissue (Fig 5A) and the mucin area per perimeter airway was quantified as a measure of goblet cell hyperplasia (GCH), a major pathological feature of asthma GCH was significantly increased in ova challenged animals versus saline controls (Fig 5B). Treatment with H_4_R antagonist, 20 mg/kg, significantly reduced GCH (vehicle 3.9 ± 0.35 versus H_4_R, 1.9 ± 0.22 pix/perimeter airway, *P *< 0.01). In agreement with its central role in goblet cell differentiation treatment with anti-IL-13 almost completely abolished antigen induced GCH (vehicle, 3.9 ± 0.35 versus anti-IL-13, 0.36 ± 0.09 pix/perimeter airway, *P *< 0.01 )

### Total lung collagen

Irregular deposition of collagen in the airways is another physiologically significant marker of Th2 cytokine mediated remodeling. Total collagen and total free collagen in homogenized lung was measured to determine the extent of antigen induced airway matrix remodeling. Treatment with both H_4_R, 20 mg/kg, and anti-IL-13 reduced free collagen levels (vehicle, 55.83 ± 2.4 μg/mg tissue versus H_4_R, 44.98 ± 2.7 μg/mg tissue *P *< 0.01, anti-IL-13, 43.49 μg/mg tissue, *P *< 0.01) (Fig 5C)

### H_4_R antagonism suppresses airway hyperreactivity

Using the sub-chronic airway protocol we did not observe significant airway hyperreactivity in vehicle treated animals over saline animals, possibly due to the extent of fibrotic remodeling in the lung (data not shown). We therefore utilized a previously reported acute model of ovalbumin induced lung inflammation to study the effects of H_4_R antagonism and IL-13 on AHR [6]. To examine airway and peripheral lung dysfunction we measured airway function in ovalbumin challenged mice upon provocation with the spasmogen, methacholine (Mch). We used a constant phase model to separate peripheral and central airway measures.

**Figure 6:**
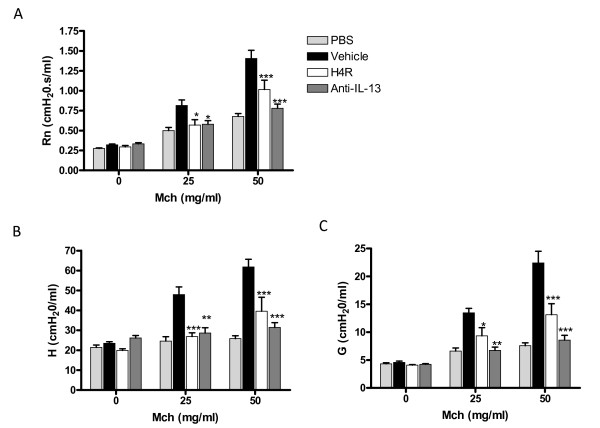
**H_4_R antagonism inhibits airway hyperreactivity and dysfunction in allergen challenged lungs**. Animals treated with vehicle, JNJ 7777120 (20 mg/kg b.i.d) and anti-IL-13 around an acute(4 ×) ovalbumin challenge were anesthetized 24 h after the last challenge and lung function measured via a small animal ventilator by forced oscillation techniques. Methacholine dose response relationships were obtained for (A) central airway resistance, (B) tissue stiffness and (C) tissue damping. Each plotted value reflects the mean values for each group of mice (n = 6-10/group). Each animal's value reflects the mean of 12 sets of data captured over a 3-minute span after each aerosol challenge was analyzed for individual animals. Significance of each treatment group compared to control vehicle-treated animals is as follows: * *P *< 0.05, ** *P *< 0.01, *** *P *< 0.001

Newtonian airway resistance, a measure of central airway resistance, was significantly increased in the vehicle animals at both 25 and 50 mg/ml Mch as compared to PBS challenged animals. Treatment with JNJ 7777120 and anti-IL-13 significantly inhibited the acute bronchoconstriction, measured as a decrease in R_N_, at both doses of Mch (Fig 6A). Similarly, lung tissue elastance (H), a measure of lung stiffness, and tissue damping (G), a putative measure of peripheral airway obstruction, were significantly inhibited by treatment of JNJ 7777120 and anti-IL-13 as compared to vehicle control animals (Fig 6B and 6C).

## Discussion

H_4_R antagonists have previously been shown to have anti-inflammatory activity when dosed prophylactically in an acute, mouse model of allergic inflammation [6]. While that study demonstrated a reduction in Th2 cytokine production, no changes in disease relevant Th2 driven pathologies were reported. In the current study we demonstrate the ability of an H_4_R antagonist to therapeutically modify existing allergic inflammation, and to attenuate airway remodeling and hyperreactivity.

The model used herein, may be considered to be mast cell independent, since sensitization protocols involving co-administration of alum with antigen have been previously demonstrated as such [6,15]. Consequently, other cells are considered to be the source of histamine acting at the H_4 _receptor in this model, sufficient to drive Th2 mediated responses. Cells including basophils, dendritic cells and neutrophil have been shown to release histamine [6,16,17], with low levels sufficient to activate the high affinity H_4_R, and H_4_R antagonists effective in mast cell deficient animals [6]. Interestingly, serotonin has also been shown to contribute to airway inflammation in mast-cell independent models [18] and has traditionally been viewed as the primary biogenic amine in rodents. A contribution of histamine and H_4_R is now demonstrated and suggests the proposed dominance of serotonin in mice to be predicated on the previous lack of effects of H1R antagonists in such models which do not block H_4_R responses [7].

We firstly demonstrated that the selective H_4_R antagonist, JNJ 7777120, was able to therapeutically reduce Th2 cytokine levels in diseased lung tissue and in response to antigen-specific re-stimulation of T cells. Furthermore, a physiologically significant role for that reduction was confirmed by the marked attenuation of IL-13 driven pathologies. Goblet cell hyperplasia and collagen deposition, classical markers of IL-13 mediated remodeling in murine models of asthma, [14,19] were strongly induced by sub-chronic allergic airway inflammation and were fully attenuated by anti-IL-13 antibody treatment. These effects were recapitulated by H_4_R antagonist treatment and in support of a direct relationship of these remodeling parameters to IL-13 levels, the extent of their amelioration by JNJ 7777120 was proportional to its reduction of IL-13 levels in the tissue.

The H_4_R and IL-13 also both appear to mediate allergic airway dysfunction. Development of airway hyperreactivity and hyperresponsiveness to innocuous stimuli is a diagnostic and pathological feature of asthma that can be recapitulated in animal models of allergic airway inflammation, and has been linked to increased airway IL-13 [20]. In our hands the model of sub-chronic airway inflammation that we utilized did not result in a reproducible increase in airway hyperreactivity, as reported by others [14]. In contrast to these studies, which used the dimensionless measure of Penh to measure AHR, and which in fact may be measuring other irrelevant respiratory changes [21], we used more reliable forced oscillation techniques to assess airway function. The absence of airway hyperreactivity observed in our model might result from an excessive remodeling and stiffening of the airways, thereby diminishing its contractile potential. Alternatively other workers have reported a 'burning out' of AHR in such chronic models [22,23]. Consequently, we utilized another well-described model to initiate airway hyperreactivity and to examine the effect of H_4_R antagonists and anti-IL-13 on this parameter.

Using this model, a robust hyperreactivity was demonstrated in vehicle treated mice as indicated by an increase in central and peripheral airways resistance. A corresponding increase in peripheral lung stiffness (elastance) was also measured in vehicle treated animals. All of these parameters were blocked both by H_4_R antagonism and anti-IL-13 treatment. Previous research has highlighted the importance of IL-13 in controlling airway hyperresponsiveness in mice [8,20]. Several studies have indicated that this is a direct effect on resident airway structural cells, and not a secondary effect due to recruitment of inflammatory cells [8,20]. Consequently, we reproduced results that supported this observation since anti-IL-13 treatment resulted in a complete abolishment of airway hyperreactivity, with no effect on airway inflammation.

Whilst these effects on goblet cell hyperplasia, collagen deposition and airway hyperreactivity supported the premise that H_4_R may modulate chronic remodeling through modulation of IL-13 production, other anti-inflammatory effects of H_4_R antagonism appear to be independent of the reduction in IL-13, since they were not recapitulated by anti-IL-13 treatment. Notably, whilst H_4_R antagonists were able to inhibit eosinophil and T cell influx into the airways, anti-IL-13 treatment did not cause a significant attenuation of these cell types. The effect on eosinophilic inflammation may in part be due to the fact that IL-5 in the airways, as measured in BALF and in lung homogenate, was reduced in H_4_R antagonist treated animals, whereas anti-IL-13 treatment had little effect. Conversely, anti-IL-13 did reduce eotaxin (CCL11) levels, whereas H_4_R treatment did not, perhaps suggesting a redundant role for eotaxin in this particular model.

The lower levels of IL-5 and IL-13 observed in the lung are likely a result of decreased recruitment of T cells since CD3+ T cells were seen to be reduced in the lung after H_4_R antagonist treatment, but not by anti-IL-13 treatment. The effect on T cell influx in to the lung may relate to the observed reduction in CCL3 and CCL5 in lung tissue which may act at both CCR1 and CCR5 to modulate T cell recruitment in to the allergic lung [24,25]. Reduction of the CCR4 ligand, CCL17 by H_4_R antagonist in a comparable study (additional data) also suggests a direct inhibition of CCR4 + Th2 cells, a sub-population implicated in asthma pathogenesis [26]. CCR1 positive T cells have been shown to be associated with IL-13 release [24] and Th2 cells are known to be the main source of IL-5 in allergic airway inflammation [27]. Of additional interest, H_4_R has been implicated in direct recruitment of T cell subsets to the lung [28] and in the release of other T cell chemoattractants such as IL-16 [29].

IL-5 and IL-13 levels in the tissue may also be reduced by a direct effect on Th2 cell cytokine elaboration. Indeed antigen restimulation of lymphocytes from H_4_R antagonist treated animals led to lower levels of both cytokines. This is likely related to the previously reported modulation of Th2 polarization by H_4_R antagonists [6]. In this previous study decreases in IL-4, IL-5 and IL-13 were also demonstrated in ovalbumin stimulated lymph node cultures from H_4_R antagonist treated or H_4_R deficient mice, despite any effect on proliferation. This effect on Th2 cytokines, via a modulation of Th2 activation, may result from a role of H_4_R in the Th2 priming capability of dendritic cells [6]. The exact mechanism for this is as yet unknown, but reduced levels of pro-Th2 cytokines such as IL-4 and IL-6 may explain the reduction in downstream Th2 polarization. Indeed, a functionally relevant reduction in IL-4 was suggested by an observed decrease in antigen-specific IgE observed with H_4_R antagonist treatment.

In contrast to findings in the acute model of asthma [6], in the sub-chronic model reported herein, antigen specific lymph node proliferation was attenuated after therapeutic treatment with JNJ 7777120. This may result from the continued activation of memory T cells in the more chronic setting, and its progressive attenuation under H_4_R blockade. One explanation of this may be the reduction in IL-4 production following restimulation seen here and in the previous model [6]. Reduction in IL-4 levels would likely suggest that subsequent Th0 to Th2 polarization of new effectors cells with each antigen challenge would be disrupted. In addition, other workers have described an H_4_R dependent reduction in Th1 promoting cytokine IL-12 production from human dendritic cells that may contribute to this effect [30]. Therefore, a possible reduction in antigen-specific Th2 cells might therefore be possible with chronic dosing of an H_4_R antagonist in a disease setting where individuals are continually exposed to allergen.

The inefficacy of anti-IL-13 on lung inflammation and tissue IL-5 levels reported here is in contrast to other reports in similar models where IL-5 was reduced in BALF by an IL-13 vaccine approach [19] or in lung homogenates, with an anti-IL-13 antibody [14]. Nevertheless our data is consistent with previous reports showing that over expression of IL-13 did not alter IL-5 expression in mouse lung [9], nor was it affected by IL-13 genetic deficiency in a mouse asthma model [20].

To put our findings into a clinical context, whilst the targeting of single cytokines, such as IL-4 [31,32] or IL-5 [33-35], has repeatedly failed to show meaningful clinical benefit in broad asthma populations a recent report has highlighted the efficacy of an inhaled, dual IL-4/IL-13 receptor blocker [36]. Consequently, a broader approach to inhibiting Th2 cytokine production, as possible with H_4_R antagonists and other small molecule inhibitors of Th2 cell polarization, may prove beneficial. Provocatively, suplatast tosilate, a small molecule modulator of dendritic cell function and of Th2 cytokine production, working through an, as yet, unknown mechanism, has demonstrated efficacy in asthmatic individuals, [37,38] with reported diminishment of IL-4 and IL-13 producing cells and concomitant goblet cell hyperplasia [39]. H_4_R antagonists share these properties, at least in mouse models examined so far.

## Conclusions

Therapeutic treatment with an H_4_R antagonist can inhibit Th2 driven pathologies such as lung remodeling in a model of sub-chronic asthma and, in addition, can improve airway dysfunction. These observations, in conjunction with the previously reported direct effects of H_4_R perturbation on mast cell and eosinophil function again reiterate the potential importance of histamine in asthma and suggest the utility of H_4_R antagonists as novel therapeutics in allergic respiratory disease.

## Competing interests

All authors are employees of Johnson & Johnson PRD, LLC.

## Authors' contributions

JMC carried out the in vivo studies, immunoassays and helped draft the manuscript. JPR performed the lung function measurements. JYM performed the histology, immunohistochemistry and analysis. RLT participated in the conception and design of the study and helped draft the manuscript. PJD conceived the study, participated in its design and coordination and drafted the manuscript. All authors read and approved the final manuscript.

## Supplementary Material

Additional file 1 **An H_4_R antagonist inhibits ova-specific IgE production**. Ova-specific IgE levels were measured in serum collected from saline, vehicle, H_4_R (20 mg/kg) or anti-IL-13 treated animals using ELISA (MD Biosciences, St.Paul, MN). n = 8-10. Significance of each treatment group compared to control vehicle-treated animals is as follows: * = p < 0.05. Click here for file

Additional file 2 **Lungs from vehicle and JNJ 7777120 treated animals were homogenized and analyzed for CCL17 (TARC) content using ELISA (R&D Systems, Minneapolis, MN) and corrected for total protein**. n = 8-10. Significance of each treatment group compared to control vehicle-treated animals is as follows: * *P *< 0.05, ** *P *< 0.01 Click here for file
