# Supplementary figures and images for: Histamine H4 receptor antagonism diminishes existing airway inflammation and dysfunction via modulation of Th2 cytokines
Source: Respir Res. 2010 Jun 24;11(1):86. doi: 10.1186/1465-9921-11-86 (PMC2914735; doi:10.1186/1465-9921-11-86)

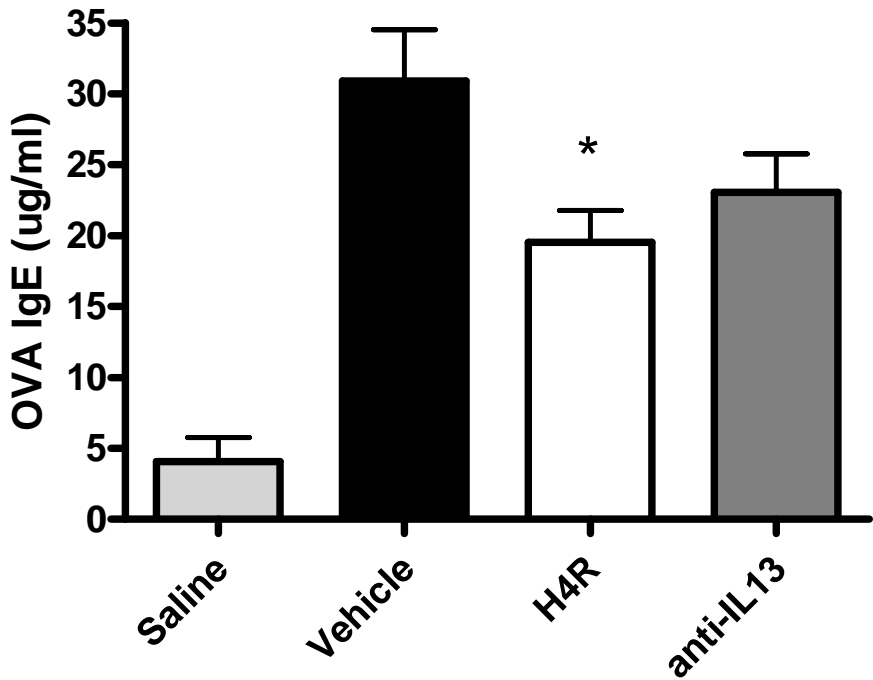

Supplement: Additional file 1 — An H4R antagonist inhibits ova-specific IgE production. Ova-specific IgE levels were measured in serum collected from saline, vehicle, H4R (20 mg/kg) or anti-IL-13 treated animals using ELISA (MD Biosciences, St.Paul, MN). n = 8-10. Significance of each treatment group compared to control vehicle-treated animals is as follows: * = p < 0.05. [file 1465-9921-11-86-S1.PDF]

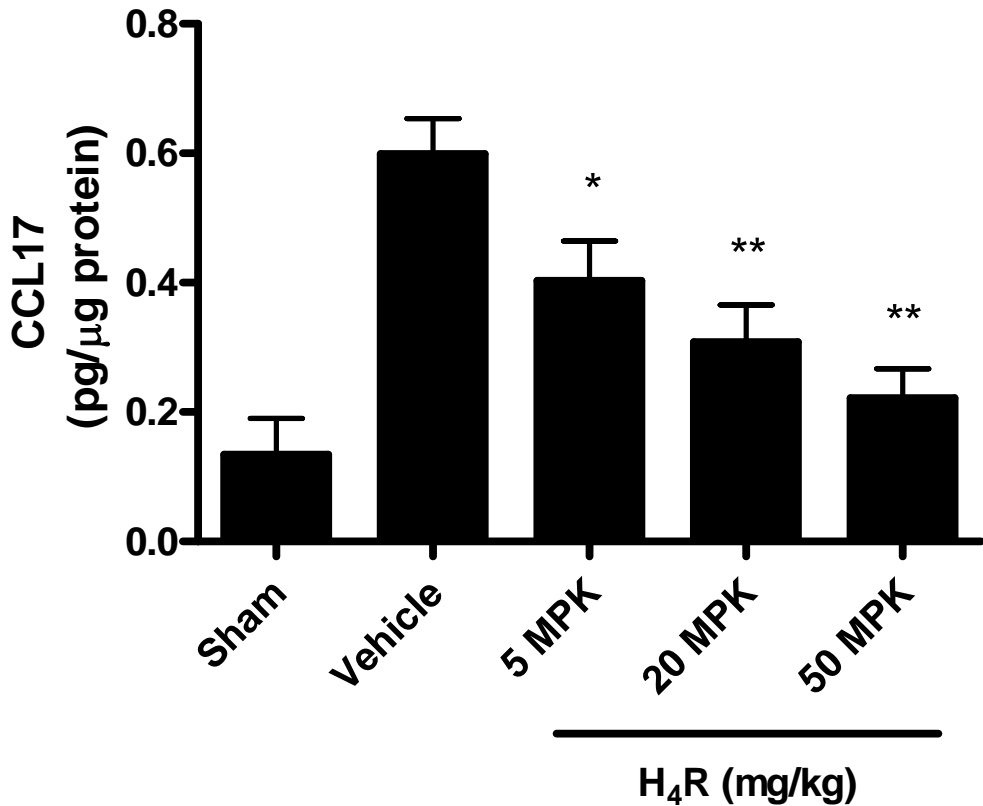

Supplement: Additional file 2 — Lungs from vehicle and JNJ 7777120 treated animals were homogenized and analyzed for CCL17 (TARC) content using ELISA (R&D Systems, Minneapolis, MN) and corrected for total protein. n = 8-10. Significance of each treatment group compared to control vehicle-treated animals is as follows: * P < 0.05, ** P < 0.01 [file 1465-9921-11-86-S2.PDF]
